# Trend and Seasonality of Hip Fractures in Catalonia, Spain: Exploring the Influence of Climate

**DOI:** 10.1007/s00223-024-01182-8

**Published:** 2024-02-10

**Authors:** Xavier Surís, Clara Rodríguez, Esteve Llargués, Maria J. Pueyo-Sánchez, Marta Larrosa

**Affiliations:** 1 Master Plan of Musculoskeletal Diseases, Department of Health, C/Travessera de les Corts, 131-159, 08028 Barcelona, Catalonia Spain; 2 Rheumatology Department, Hospital General de Granollers, Granollers, Spain; 3 School of Medicine and Health Sciences, Universitat Internacional de Catalunya, Sant Cugat del Valles, Spain; 4 Fundació Institut Universitari per a la Recerca a l’Atenció Primària de Salut Jordi Gol i Gurina (IDIAPJGol), Barcelona, Spain; 5 Facultat de Biologia, Universitat de Barcelona, Barcelona, Spain; 6 Internal Medicine Department, Hospital General de Granollers, Granollers, Spain; 7 Assistance and Participation Area. La Unió, Association of Health and Social Entities, Barcelona, Spain

**Keywords:** Hip fracture, Epidemiology, Incidence rate ratio, Seasonal presentation, Osteoporosis, Climate, Weather, Radiation

## Abstract

To describe the secular trend and seasonality of the incidence of hip fracture (HF) and its relationship with climatic variables during the period 2010–2019 in Catalonia in people aged ≥ 65 years. The results were analyzed by sex, age groups (65–74, 75–84, and ≥ 85), and types of fracture (extracapsular and intracapsular). Data on sex, age, type of fracture, year, and month of hospitalization of patients admitted with a diagnosis of HF between January 1, 2010 and December 31, 2019 were collected. Crude and standardized HF incidence (HFi) rates were obtained. Data on the monthly mean of climatological variables (temperature, insolation, icy days, rain, relative humidity, atmospheric pressure, and wind force) were obtained from the network of meteorological stations in Catalonia. Time series analytical statistics were used to identify trends and seasonality. Linear regression and a seasonal autoregressive integrated moving average (ARIMA) were used to analyze the relationship of each climatic parameter with fracture rates. In addition, generalized additive models were used to ascertain the best predictive model. The total number of HF episodes was 90,149 (74.1% in women and 25.9% in men). The total number of HFs increased by 6.4% between 2010 and 2019. The median age (SD) was 84.5 (7.14) and 54% of patients were ≥ 85 years of age. Extracapsular fractures were the most common (55%). The standardized incidence rates decreased from 728.1/100,000 (95% CI 738.6–769.3) to 624.5/100,000 (95% CI 648.7–677.0), which represents a decrease of 14.2% (*p* < 0.05). The decline was greater at older ages. There were seasonal variations, with higher incidences in autumn (27.2%) and winter (25.7%) and lower rates in summer (23.5%) and spring (23.6%). Seasonality was more pronounced in elderly people and men. In the bivariate regression analysis, high temperatures and greater insolation were negatively associated with the HF rate, while the number of icy days, rainy days, and high relative humidity were associated with a higher incidence of fractures in all age groups and sexes. In the regression analysis using the seasonal ARIMA model, only insolation had a consistently significant association with overall HFi, after adjusting by trend and other climatic parameters. While the global number of HFs grew in Catalonia due to increases in the elderly population, the standardized HF rate decreased during the years 2010–2019. There was a seasonal trend, with predominance in the cold months and correlations with climatic parameters, especially with insolation.

**Supplementary Information:**

The online version contains supplementary material available at 10.1007/s00223-024-01182-8.

## Introduction

According to The Global Burden of Diseases, Injuries, and Risk Factors Study (GBD), approximately 178 million new fractures occurred worldwide in 2019 [1]. Hip fractures are the leading cause of health expenditures among all fragility fractures (FF) in the European Union [2]. The overall number of HFs is increasing in developed countries due to aging populations. Nevertheless, the secular trend of HFi varies in different countries; while data from Australia [3], Canada [4], and Europe [5–7] shows a decreasing trend, the scarce data from Asia and South America (with the exception of Hong-Kong and Taiwan) point to a rising trend [8]. In addition, there is a wide variation in the incidence of HF between countries and across different geographical regions. The highest rates were found in Europe and North America and especially in the Scandinavian countries [9].

Among the many factors that can influence the incidence and prevalence of HF, some are related to climate and latitude, with the highest incidences occurring in high latitude countries [10]. Furthermore, a relationship between HFi, lower latitude, and exposure to ultraviolet (UV) light has been described in Sweden [11]. Moreover, most of the evidence points to a seasonal pattern in HFi, with higher rates occurring during cold months in most countries [12]. In Spain, which has a Mediterranean climate, higher rates of HF show a prominent seasonality, especially in autumn, and particularly in older populations [13, 14].

Two main hypothesis have been put forward to explain the relationship between climate change during the year, latitude, and the global variations in HFi. The first implies that lower exposure to sunlight in autumn and winter, especially in in high latitude countries, could cause lower vitamin D synthesis, which has been linked to secondary hyperparathyroidism, osteoporosis, and osteomalacia, with an increased risk of fracture [15, 16]. On the other hand, associations with other meteorological factors such as low temperatures, freezing rain, and snow would be due to an increased risk of falls. All of these meteorological parameters have been investigated in different environments with conflicting results, due to differences in geographical locations, study periods, and the variety of analytical methods employed [12, 17].

Spain has a public health system with universal coverage that collects and manages health data from most of the population. In addition, Catalonia, a community in northeast Spain, has a very extensive network of meteorological stations distributed throughout the territory that automatically record data on numerous daily climate variables, including the intensity of solar radiation. Therefore, our objective was not only to describe the trend and the seasonality of HFi, but also to investigate its association with climatological variables over an extended period in different sexes and age ranges between 2010 and 2019. Although climate is not a modifiable factor, understanding how meteorological variables might affect the risk of fractures at different ages and seasons could help us develop preventive strategies and may provide clues as to how climate change may affect the epidemiology of fractures in the future.

## Materials

### Data Sources

A time series analysis of HF in Catalonia, a community in northeast Spain with 7.9 M inhabitants, was carried out with data collected from the Minimum Basic Data Set, a register composed of all acute care hospital discharges. The diagnoses were coded according to the International Classification Disease 9th Edition (ICD-9-CM) from 2010 to 2017 and the 10th Edition (ICD-10-CM) in 2018 and 2019. Data on sex, age, type of fracture, and date of hospitalization were collected from people aged ≥ 65 years whose primary or secondary diagnosis was a HF (codes 820.0/820.2/S72.0/S72.1/S72.2) between January 1, 2010 and December 31, 2019. We decided not to use data after 2019 since the COVID-19 pandemic had an extraordinary impact on fracture rates [18]. Patients with open fractures, those living in communities outside of Catalonia, and readmissions were excluded. HFs were classified according to the anatomical location as intracapsular (subcapital and basicervical fractures) and extracapsular (pertrochanteric and subtrochanteric) fractures. Direct age-standardized rates were estimated using the Catalan population in 2014 as a baseline. Population data were obtained from the Insured’s Central Register of Catalonia. Using the hospitalization date, we grouped the months as follows: winter (January, February, and March), spring (April, May, and June), summer (July, August, and September), and autumn (October, November, and December). To compare the HFi for different periods of the year, the monthly number of fractures was adjusted to 30 days and the seasons to 91.25 days.

**Fig. 1:**
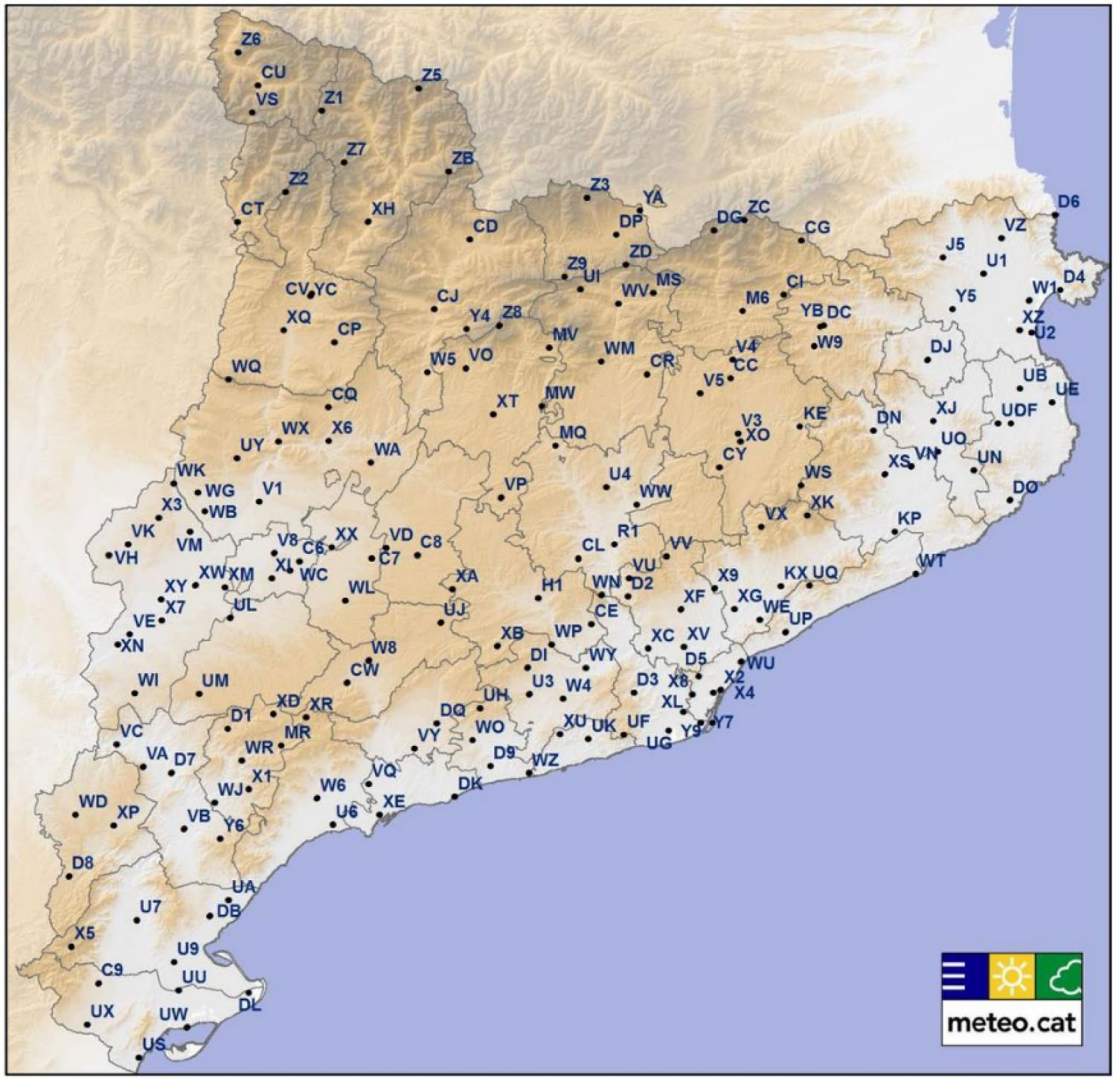
Map of Catalonia showing the geographical distribution of the meteorological stations in 2016

Data on the meteorological variables were obtained from the network of automatic weather stations operated by the meteorological service of Catalonia (Meteocat). This network was created in 1996 and integrates all the automatic meteorological stations managed by Meteocat and distributed throughout Catalonia (Fig. 1). Of 210 stations belonging to this network, we obtained data from 176 stations with an altitude of < 1500 m, since no inhabited areas above this altitude are to be found in Catalonia. Latitude in the Catalonia ranges between the northernmost point, at 42° 50′, and the southernmost, at 40° 32′. This slight difference in latitude is irrelevant from a climatic point of view. The climate of Catalonia (with the exception of some mountainous regions) is Mediterranean, with mild temperatures in winter and hot and dry summers. Catalonia has a high insolation level (between 2000 and 2600 h a year) and snow and ice remain very isolated phenomena. Data included were as follows: monthly average temperature (Celsius degrees), wind speed (m/s), relative humidity (%), daily global solar radiation (Megajoules/m^2^), and atmospheric pressure (Hectopascals). We also calculated the monthly number of icy days (days with temperatures < 0 °C), as well as the number of days with precipitation (those with a minimum average of 0.2 mm/m^2^). The measurement of global solar radiation refers to irradiance on a horizontal surface and represents direct and diffuse incident solar radiation. It is measured in the wavelength range of 0.3 to 3 μm, which corresponds to the spectrum ranging from UV to infrared.

### Statistical Analysis

#### Data Description

Quantitative data are described by mean and standard deviation (SD) or median and interquartile range (IQR). A Student’s* t* test was used to assess differences between means. After confirming that age followed a non-normal distribution (Shapiro–Wilk test for normality), we used the non-parametric Mann–Whitney test to investigate age differences between periods of time and types of fractures. Categorical variables, described by frequencies and percentages, were compared using the chi-square test.

#### Hip Fracture Incidence and Trend

Annual crude incidence rates (number of fractures per 100,000 inhabitants) with 95% confidence interval (95% CI) over the 10 year study were calculated for the entire population assuming a Poisson distribution. Subanalysis by sex and age groups (age intervals were set at 65–74 years, 75–84 years, and 85 and above) and anatomical fracture types (intracapsular and extracapsular) was performed. Direct age standardization was determined for the entire population and by sex using the age distribution of the Catalan population in 2014 as standard population. A time series of standardized HFi ratios, as well as the monthly mean of each meteorological parameter in 120 consecutive months, was established. A time series may consist of various components that must be isolated in order to analyze their relationship with other variables. The first component is the trend, which is an irregular long-term movement in the time series lacking any calendar effects. The trend defines the growth or decline of the time series. The second component, seasonal variation, is a regular pattern of change recurring over time. The seasonal component consists of effects that are stable with respect to time, direction, and magnitude, and shows a pattern that is repeated after “s” observations. The third is the residual, an irregular component that remains after the seasonal and trend components have been removed. It represents the non-predictable result of short-term fluctuations. To analyze this trend, the Seasonal Mann–Kendall test was used to assess whether the series of HF rates had changed over time. Both the Mann–Kendall test and Sen’s slope are widely used for detecting statistically significant trends and determining their magnitudes, respectively, in a time series.

#### Seasonality and Association of HFi with Meteorological Parameters

The seasonality of the HF series and its relationship with the climatic parameters were examined using different analytical strategies. HFi rate ratios with 95% CI were calculated for each season of the year. Associations between meteorological parameters and HFi rates were first analyzed using Pearson correlations. In addition, to identify and analyze the individual components of the HFi time series, a seasonal ARIMA model was built. The general multiplicative form of seasonal ARIMA (*p*, *d*, *q*) × (*P*, *D*, *Q*) contains the non-seasonal autoregressive parameters *p* and *q*, the seasonal autoregressive and moving parameters *P* and *Q*, and two non-seasonal and seasonal differencing orders designated *d* and *D*. We selected the type and order of the parameters which were better adjusted to the time series through the autoarima function**.** Dickey–Fuller and Kruskal–Wallis statistics were used to assess the non-stationarity nature of the time series. ARIMA regression analysis, which combines regression analysis with time series modeling, was used to ascertain how each of the climate parameters (predictor variables) was associated with HFi in the overall population and each subgroup, after adjusting by time trend. This combined model aimed to provide a comprehensive understanding of the relationship between time series variables and predictors.

Finally, a generalized additive model (GAM) was used to study the effects of combined climate variables on HFi rates. GAM models were built by adding meteorological parameters to the year and month as predictor variables in order of higher to lower correlation with HFi rates. A GAM model permits the inclusion of more independent variables in a parametric linear model, and is useful when the different parameters are highly interactive (as is the case with meteorological variables). The threshold for statistical significance was set at a 2-sided *α*-value of 0.05. All analyses were carried out using R v4.0.3. This study used retrospective anonymized data from the Minimum Basic Data Set. The study complied with the ethical guidelines of the Declaration of Helsinki. For this type of study, formal consent is not required.

## Results

Over the 10 year study period, total HF episodes numbered 90,149 (74.1% in women and 25.9% in men). The number of observed fractures increased from 8741 in 2010 to 9297 in 2019. The female-to-male ratio decreased significantly from 2.96 (2010) to 2.69 (2019) (*p* < 0.01). Mean age (SD) was 84.5 (7.1); women 84.8 (7.0), men 83.4 (7.5) (*p* < 0.0001), and increased from 83.9 (6.9) years in 2010 to 84.9 (6.4) years in 2019 (*p* < 0.0001). The population aged 65 years and older in Catalonia increased from 1.25 M to 1.43 M people, a 14.8% increase, with the oldest group (≥ 85 years) increasing 39%. This age group represented 14% of the population in 2010 and 16.9% in 2019. Table 1 describes the number and percentage of fractures by sex, season of the year, bone localization, and calendar year in both the overall population and in the different age groups. The percentage of fractures corresponding to the oldest group (≥ 85 years) increased from 49.2% in 2010 to 58.3% in 2019. Extracapsular fractures were the most frequent (55%) in the overall population, but not in those under 75 years (48.4%). Thus, subjects with extracapsular fractures were significantly older than those with intracapsular fractures (*p* < 0.0001). The extracapsular-to-intracapsular ratio remained stable throughout the 10 year period. The mean temperature over the 10 year period was 14.3 °C, with a minimum seasonal average of 7.6 °C in winter and a maximum average of 22.1 °C in summer. Mean insolation was higher in spring and lower in autumn. Mean monthly icy and rainy days were higher in winter and autumn, respectively (Table S1 in Supplementary Material).

**Table 1:** Number and percentage of fractures by sex, season of the year, hip localization, and calendar year in overall population and different age groups

Age	65–74 years	75–84 years	> 84 years	Overall
Number of fractures	9016	32,455	48,678	90,149
Sex				
Women	5759 (63.9%)	23,756 (73.2%)	37,320 (76.7%)	66,835 (74.1%)
Men	3257 (36.1%)	8699 (26.8%)	11,358 (23.3%)	23,314 (25.9%)
Season				
Winter	2276 (25.2%)	8331 (25.7%)	12,524 (25.7%)	23,131 (25.7%)
Spring	2140 (23.7%)	7842 (24.2%)	11,305 (23.2%)	21,287 (23.6%)
Summer	2241 (24.9%)	7592 (23.4%)	11,350 (23.3%)	21,183 (23.5%)
Autumn	2359 (26.2%)	8690 (26.8%)	13,499 (27.7%	24,548 (27.2%)
Type of fracture				
Intracapsular	4656 (51.6%)	15,133 (46.6%)	20,792 (42.7%)	40,581 (45.0%)
Extracapsular	4360 (48.4%)	17,322 (53.4%)	27,886 (57.3%)	49,568 (55.0%)
Calendar year				
2010	849 (9.4%)	3592 (11.1%)	4300 (8.8%)	8741 (9.7%)
2011	775 (8.6%)	3435 (10.6%)	4341 (8.9%)	8551 (9.5%)
2012	765 (8.5%)	3459 (10.7%)	4472 (9.2%)	8696 (9.6%)
2013	829 (9.2%)	3429 (10.6%)	4597 (9.4%)	8855 (9.8%)
2014	844 (9.4%)	3168 (9.8%)	4723 (9.7%)	8735 (9.7%)
2015	951 (10.5%)	3327 (10.3%)	5073 (10.4%)	9351 (10.4%)
2016	971 (10.8%)	3285 (10.1%)	5275 (10.8%)	9531 (10.6%)
2017	979 (10.9%)	2997 (9.2%)	5389 (11.1%)	9365 (10.4%)
2018	1039 (11.5%)	2902 (8.9%)	5086 (10.4%)	9027 (10.0%)
2019	1014 (11.2%)	2861 (8.8%)	5422 (11.1%)	9297 (10.3%)

### Hip Fracture Incidence and Trend

**Fig. 2:**
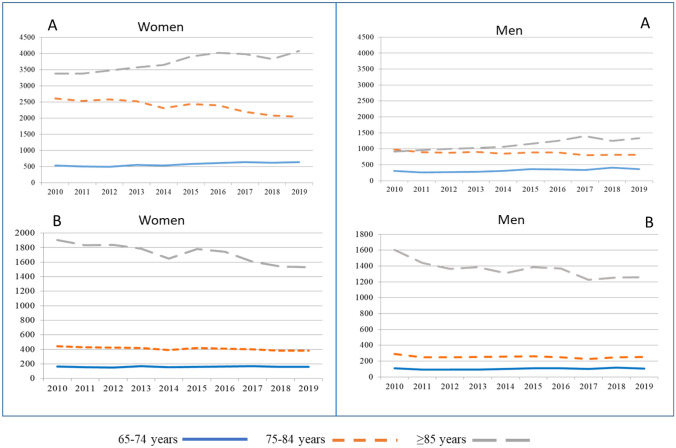
Crude number of HFs by age and sex (**A**) and age-standardized HFi by age and sex (**B**) in the Catalan population from 2010 to 2019

Overall annual crude HFi rates per 100,000 people decreased from 697.7 (95% CI 683.1–712.4) in 2010 to 646.5 (95% CI 633.3–659.6) in 2019, representing 7.3% decrease. By sex, the rates decreased more in women than in men and in people aged 75 to 84 years than in the oldest group, with no significant decline in those aged < 75. The extracapsular fracture rate decreased 6.9%, while that of intracapsular fractures decreased 8.0%, both significant. Age-standardized incidence rates decreased from 728.1 (95% CI 713.6–742.5) to 624.5 (95% CI 611.1–637.8) in the overall population, representing a 14.2% decrease (Tables S2 and S3 in Supplementary Material). Figure 2 shows the trend of the number of HFs and HFi by sex and age group. Age-standardized HFi showed a downward trend over the ten-year period, with a temporary increase in 2015–2016. Using the Seasonal Mann–Kendall test for the 120 monthly time series data of HFi in the overall population, there was an downward trend (*p* = 0.003, Sen’s slope; − 0.41), which was also observed in women (*p* < 0.001, Sen’s slope; − 0.94), but not in men. Age-adjusted HFi showed a declining trend in the 75–84 year group (*p* < 0.001, Sen’s slope − 1.15) and in people over 84 years of age (*p* = 0.027, Sen’s slope − 1.44), although not in those aged 65–74. Both age-standardized intracapsular (*p* = 0.008, Sen’s slope − 0.23) and extracapsular fractures (*p* = 0.006, Sen’s slope − 0.24) showed declining trends.

### Seasonality and Association of HFi with Meteorological Parameters

**Fig. 3:**
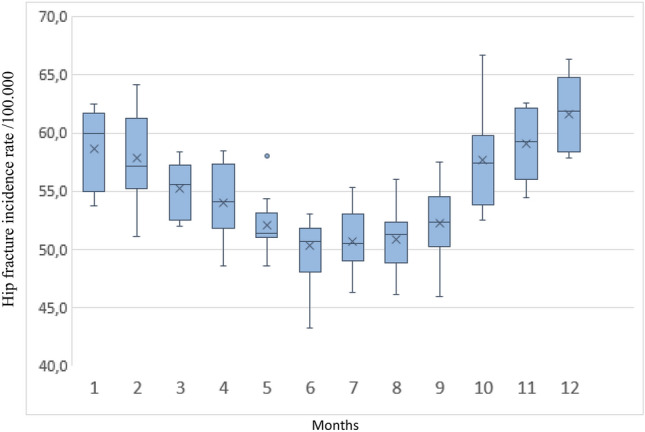
Monthly differences in HFi in Catalan population from 2010 to 2019 (months adjusted to 30 days). The boxes represent the interquartile range, the horizontal lines within the boxes represent the median values, the cross represents the mean value, and the whiskers represent minimum and maximum values

**Fig. 4:**
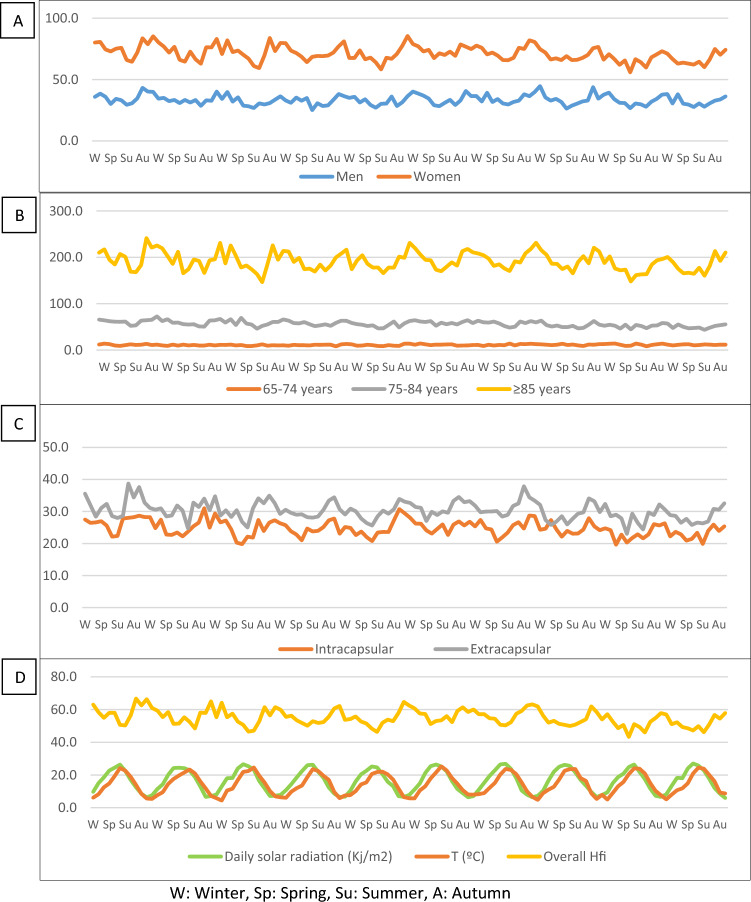
HFi × 100.000 in people ≥ 65 years, by sex (**A**), age groups (**B**) and type of fracture (**C**). Overall HFi with average temperature and monthly solar radiation. 120 consecutive months adjusted to 30 days

Monthly mean (SD) HFi after adjusting months to 30 days was 55.0 (4.9) for the whole series; 58.7 (3.4) in January; 57.9 (4.0) in February; 55.3 (2.3) in March; 54.0 (3.3) in April; 52.1 (2.5) in May; 50.3 (3.8) in June; 50.7 (2.8) in July; 50.8 (2.8) in August; 52.3 (3.4) in September; 57.7 (4.3) in October; 59.1 (3.1) in November; and 61.6 (3.2) in December. These figures were higher in cold months, where greater variability was also observed (Fig. 3). Figure S1, in the Supplementary Material shows a similar pattern by sex, age group, and type of fracture, except in people aged ≤ 74 years. In those over 84 years of age, there was a marked increase between September and October. After adjusting the seasons to 91.25 days, 25.7% of HFs occurred in winter, 23.6% in spring, 23.5% in summer, and 27.2% in autumn. Taking summer as the reference, there was a significant average increase of 15.9% in the HF incidence in autumn, 11.6% in winter, and 1.6% in spring. By sex, the percent increase in autumn with respect to summer was 17.4% in men; IRR (95% CI) 1.17 (1.14–1.21) and 15.5% in women; IRR (95% CI) 1.15 (1.14–1.17). The female-to-male ratio was higher during the warm seasons (2.95 in spring, 2.93 in summer) than in the cold seasons (2.88 in autumn, 2.73 in winter). By ages, the differences between summer and autumn were higher in the oldest group (18.9%). Extracapsular fractures showed a higher change between seasons than intracapsular fractures (Table 2). Figure 4 shows the seasonal pattern of HFi in the overall population, as well as by sex, age ranges, and fracture types. Greater seasonality can be observed in older people and in extracapsular fractures, which are more common in this age range. A clear inverse correlation can be seen between the fracture rate and the insolation and temperature curves. The ascending curve representing sunshine is ahead of that of temperature, given that in most years, the maximum solar radiation occurred in June and the maximum temperature in July (Fig. 4D). June is the month with the lower HFi rate and the higher average insolation, while December had the higher HFi rate and lower average insolation. The average temperature peak occurred after the minimum value in HFi, while the lowest temperature occurred in January, after the peak incidence in HF (Table S4 in Supplementary Material). In the bivariate analysis, higher insolation and temperature showed a negative correlation with HFi in both sex and fracture type and in all age groups. Mean monthly icy days, average relative humidity, and average days with precipitation showed a significant positive association, while no correlation was found with atmospheric pressure or average wind speed. All of the parameters positively or negatively associated with HFi showed an ascending gradient from the youngest to the oldest age group (Table 3).

**Table 2:** Seasonal average HF incidence rates by sex, age groups, and type of fracture. Difference between autumn and summer

	Winter	Spring	Summer	Autumn	Difference autumn/summer	
					IRR	%
Sex						
Overall						
Mean	174.47	158.80	156.30	181.13	1.16	− 15.9
LCI	172.24	156.67	154.19	178.86	1.15	
UCI	176.70	160.92	158.41	183.40	1.17	
Women						
Mean	222.23	206.42	202.70	233.86	1.15	− 15.4
LCI	218.91	203.22	199.53	230.46	1.14	
UCI	225.55	209.62	205.87	237.27	1.17	
Men						
Mean	109.55	94.12	93.28	109.50	1.17	− 17.4
LCI	107.22	91.93	91.10	107.17	1.14	
UCI	112.65	96.97	96.12	112.60	1.21	
Age						
65–74 years						
Mean	34.84	32.39	33.55	35.32	1.05	− 5.3
LCI	33.41	31.02	32.16	33.89	1.02	
UCI	36.26	33.76	34.95	36.75	1.08	
75–84 years						
Mean	178.94	166.58	159.52	182.59	1.14	− 14.5
LCI	175.12	162.90	155.92	178.74	1.13	
UCI	182.75	170.26	163.12	186.44	1.16	
≥ 85 years						
Mean	605.41	540.48	536.73	638.35	1.19	− 18.9
LCI	594.91	530.55	526.84	627.57	1.18	
UCI	615.91	550.40	546.62	649.13	1.20	
Type of fracture						
Extracapsular						
Mean	95.03	87.34	85.85	100.54	1.17	− 17.1
LCI	93.38	85.76	84.28	98.85	1.16	
UCI	96.68	88.92	87.42	102.24	1.18	
Intracapsular						
Mean	79.44	71.46	70.45	80.59	1.14	− 14.4
LCI	77.93	70.03	69.03	79.07	1.13	
UCI	80.95	72.89	71.87	82.11	1.16	

*IRR* incidence rate ratio, *LCI* lower confidence interval, *UCI* upper confidence interval

**Table 3:** Correlation between average monthly meteorological variables and hip fracture incidence in the bivariate regression analysis in Catalonia 2010–2019

	Insolation (MJ/m2)	Temperature (ºC)	Rainy days (number)	Frosting days (number)	Relative humidity (%)	Atmospheric pressure (hPa)	Wind speed (m/s)
Sex							
Overall population	− 0.736***	− 0.698***	0.264**	0.616***	0.449***	0.007	0.096
Men	− 0.688***	− 0.705***	0.220*	0.638***	0.410***	0.029	0.167
Women	− 0.673***	− 0.613***	0.255**	0.533**	0.419***	0.004	0.050
Type of fracture							
Extracapsular	− 0.657***	− 0.662***	0.222**	0.584***	0.364***	0.043	0.114
Intracapsular	− 0.687***	− 0.619***	0.259*	0.547***	0.451***	− 0.026	0.045
Age groups							
65–74 years	− 0.206*	− 0.216*	0.091	0.264**	0.115	− 0.020	0.148
75–84 years	− 0.572***	− 0.583***	0.272**	0.490***	0.330***	− 0.100	0.124
≥ 85 years	− 0.732***	− 0.661***	0.211*	0.583***	0.463***	0.088	0.031

***p < 0.001, **p < 0.01, *p < 0.05

Figure S2 in the Supplementary Material shows the decomposition of the additives time series, the seasonal component and the residuals in the seasonal ARIMA analysis. The residuals of the model were considered white noise, as the graph showed fluctuations around zero, which was confirmed with the Dickey–Fuller test (*p*-value < 0.05). The Kruskal–Wallis test confirmed the non-stationarity of the time series (*p*-value = 0.0003). In the multivariate time series analysis, after adjusting for time trend and the rest of the climate parameters, the average monthly insolation was the meteorological parameter that showed the highest (negative) association with HFi both overall and across all subgroups, excepting people aged 65–74 years. This association was very pronounced in people aged ≥ 85 (*β* = − 2.72). Relative humidity showed a marginal negative association in the overall population, in women and in people over 75 years. Average number of rainy days only showed a positive association in people aged 75–84 years, while atmospheric pressure was positively associated with HFi in men. The monthly mean temperature, icy days, and wind speed did not show any significant correlation after accounting for trend and the rest of the climate variables (Table 4).

**Table 4:** Results of the ARIMA analysis

	Overall HFi			Men			Women			65–74 years				75–84 years				≥ 85 years		
	*β*	SE	*p*	*β*	SE	*p*	*β*	SE	*p*	*β*	SE	*p*	*β*		SE	*p*	*β*		SE	*p*
Daily insolation (MJ/m^2^)	− 0.535	0.132	** < 0.001**	− 0.281	0.116	**0.015**	− 0.79	0.185	** < 0.001**	− 0.001	0.064	0.983	− 0.514		0.159	**0.001**	− 2.72		0.429	**0.000**
Temperature (ºC)	− 0.092	0.134	0.493	− 0.174	0.104	0.096	0.003	0.189	0.988	0.062	0.061	0.311	− 0.184		0.153	0.229	− 0.099		0.447	0.824
Rainy days (number)	0.148	0.13	0.255	0.057	0.136	0.675	0.301	0.188	0.11	− 0.025	0.075	0.739	0.409		0.184	**0.026**	0.656		0.707	0.354
Frosting days (number)	0.114	0.127	0.369	0.086	0.116	0.46	0.08	0.181	0.66	0.1	0.062	0.105	0.053		0.157	0.737	0.003		0.544	0.995
Relative humidity (%)	− 0.243	0.121	**0.045**	− 0.101	0.13	0.437	− 0.406	0.176	**0.021**	0.068	0.07	0.328	− 0.368		0.169	**0.03**	− 1.284		0.563	**0.023**
Atmospheric pressure (hPa)	− 0.024	0.101	0.809	0.048	0.012	** < 0.001**	0.027	0.144	0.851	− 0.004	0.053	0.936	− 0.056		0.133	0.672	0.571		0.47	0.224
Wind speed (m/s)	− 0.416	1.462	0.776	0.121	1.291	0.925	− 0.399	2.174	0.854	1.329	0.812	0.101	-2.27		1.969	0.249	− 6.078		6.022	0.313

Association of the monthly average of meteorological variables with HFi in the general population, by sex and age groups after adjusting for trend and meteorological parameters. The beta coefficient represents the estimated change in monthly HFi for a change of one unit in the meteorological predictive variable, holding all other predictors constant

*SE* standard error

Numbers in bold mean significant results

Results of the GAM model showed that the addition of insolation + temperature (*R*^2^ = 0.78) proved slightly higher to insolation alone (*R*^2^ = 0.76). When relative humidity, number of icy days and rainy days were added, the prediction model with the highest level of association was achieved, but revealed only a marginal improvement (*R*^2^ = 0.79). Figure S3, Supplementary Material, represents an evaluation of the model fitting showing a good accuracy in its predictive ability. The other variables were not included in the model, as they were not significant in the bivariate analysis.

## Discussion

In this extensive 10 year analysis combining HF and meteorological data, a decline in HFi among people aged ≥ 65 years in Catalonia, Spain, was observed from 2010 to 2019. There was a distinct seasonal pattern, with higher rates in autumn and lower rates in summer, with more pronounced fluctuations in the older age groups. Solar radiation exhibited a robust negative correlation with HFi, higher than that of temperature.

### Hip Fracture Incidences and Trends

While the elderly population has steadily increased in Catalonia over the last decade, the number of HFs has not risen at the same rate. These data confirm the tendency of a decrease in the standardized incidence in our [7, 19, 20] and other, mainly Western, countries [3, 21–23]. The decreasing rates in most countries in Europe, North America, and Oceania, observed since the last decade of the twentieth century, differs from the increasing incidences observed in most Asian countries [8]. The combination of several factors could explain this downward trend: sociodemographic changes such as historical cohort effects have been described in Spain, which would also influence the differences in incidence and trends between eastern and western communities [24]. Moreover, improvement in healthy habits such as increasing physical activity [25] and cessation of tobacco consumption [26] have been related to a decrease in HF risk in postmenopausal women. In that sense, over the last decades some interventions have been implemented in Catalonia to increase the proportion of adults complying with physical activity recommendations [27] and decreasing smoking habits [28]. Whether or not due to these health policies, the prevalence of healthy levels of physical activity has risen and the percentage of tobacco consumption has decreased, while obesity, a protective factor [29], has climbed [30]. Regarding the possible relationship between the use of anti-osteoporosis medications and the trend in HFi, the beginning of our time series coincides with the concerns raised due to the association of bisphosphonates with atypical fractures, which led to a dramatic decrease in the use of those medications in Spain [31]. However, to better understand the influence of anti-osteoporosis medications on the HFi trend, it would be necessary to analyze other data, such as adherence rates, the baseline risk of fractures in the treated population, and the comparative effectiveness of medications used in different periods of time. Secondary prevention programs (Fracture Liaison Services) in line with International Osteoporosis Foundation guidelines have had an increasing impact in our country in recent years. These units have been shown to improve the outcomes of patients with FF and to reduce fracture risk [31]. Finally, climate change, with increasing temperatures over time, could have influenced HFi to some extent. However, based on our results, the HFi would decrease approximately by 1% for every 1 °C increase in temperature. Given that air temperature is increasing 0.25 °C every decade in Catalonia [32], the expected decrease in HFi due to rising temperatures over a ten-year period would be 0.25%, which is insignificant compared to the observed one.

By sex, the standardized HFi rate decreased more in women than in men. This has been described in ours and other countries and could be related, in part, to a lower awareness in the diagnosis of osteoporosis and the prescription of preventive drugs in men [33]. There were also some differences between age groups; a significant downward trend in the age-specific incidence ratio was found in people ≥ 75 years, while it was more pronounced from 75 to 85 years than in people aged ≥ 85. The downward trend previously described in people ≤ 75 years up to 2014 in Catalonia [19] was lost.

### Seasonality and Association of HFi with Meteorological Parameters

HF rates were clearly affected by seasonal variations, with a higher prevalence in cold seasons and a more pronounced effect in older people and in men. The average daily solar radiation in our series was 16.2 MJ/m^2^, ranging from a minimum of 9.0 MJ/m^2^ in autumn to a maximum of 22.8 MJ/m^2^ in spring. According to our ARIMA seasonal regression analysis, for every MJ/m^2^ increase in average daily solar radiation, the monthly HFi rate × 100,000 decreased by 2.72 points in people over 84 years of age. Given that the average monthly HFi rate for people in this age group is 191.3, this represents a reduction of 1.42% for every MJ/m^2^. The same increase in solar radiation was only associated with a 0.92% decrease in HFi in people aged 75 to 84 years. Consequently, the lower levels of radiation in the colder months may have had a more pronounced impact on older people, due to a decrease in the skin's ability to produce vitamin D with age [34, 35]. Besides, colder temperatures can heighten the risk of falls by affecting neurosensory abilities [36, 37]. In our time series, autumn was the season with the lowest average insolation and with the highest global HFi in the overall population and among women. Conversely, men exhibited the highest HFi during winter, the coldest season. The seasonal pattern was more pronounced in men than in women, with women-to-men ratio much lower in winter and higher in spring. Differences between sexes in the seasonality of HF have been described, although only in some geographic areas. In a study focused on New York City, seasonality had a more marked effect in men than in women, with a greater susceptibility to HF in men during cold months. The greater propensity for men to go outdoors in worse weather conditions was mentioned as a possible explanation [38]. Otherwise, other cohorts from Canada and Taiwan found no differences between age groups or sexes [21, 39]. The autumn predominance of the HFi is the most frequently observed pattern in Spain [13, 14]. In a geographically close cohort from the Mediterranean region in Spain, in which the day was used as a time unit, the overall pattern of seasonality was similar to ours. However, the authors’ findings differed in that a close relationship between HFi and wind intensity was found, especially in younger people [13]. A possible explanation comes from the different time units used in the analyses. When the day is used as a unit of time, meteorological factors that increase the probability of falls, such as wind, snow, or ice, could be better evaluated. In contrast, when the month is used, those climatic variables that have a more long-term effect would be more apparent in the results.

Solar radiation, followed by temperature, were the two climate variables that exhibited a strong and negative association with HFi in the bivariate analysis. Moreover, in the seasonal ARIMA regression analysis, insolation, but not temperature, retained a statistically significant association with HFi. Two systematic reviews have examined the relationship between cyclical changes in fracture rates and climatic parameters, revealing a protective effect of higher temperatures [12, 17]. One of these reviews, specifically focused on HFs, also summarized prior studies investigating the influence of sunshine on HF risk. These studies, generally using monthly aggregates, consistently showed a negative association [12]. Those studies estimated insolation based on a timed measure of sun exposure (in minutes, hours, or days). However, the amount of solar radiation on earth not only depends on the time of solar exposure, but also on other factors, such as the hour of the day, the season of the year, the ozone layer, the surface reflection, the altitude, and the latitude [41]. Our analysis is based on the measurement of direct and diffuse incident solar radiation measured at a wavelength that corresponds to the spectrum spanning UV to infrared. As the cutaneous synthesis of vitamin D depends on the amount of UVB light, we believe that our estimate is more reliable than those based only on hours of sunshine. Moreover, the design of our analysis, based on monthly data, is better suited to examine the effects of insolation on fracture incidence. While the impact of sun exposure on vitamin D synthesis from pre-vitamins is rapid [40], its beneficial effects on bone resistance are gradual. A relationship between cumulative UV exposure, bone mineral density and the risk of falls and fractures has already been documented [41]. In contrast, another study conducted in inland Spain found a negative association between UVB light radiation and HFi in the short term. The authors hypothesized that increased sunshine could also exert its effect through enhanced visibility, thereby reducing the risk of falls [14]. A work performed in Boston, MA (USA), a city with the same latitude as Catalonia, showed that from November to February there was insufficient UBV radiation to synthesize previtamin D from 7-dehydrocholeterol. Beginning in March; however, the skin was capable of synthesizing vitamin D [42]. In a study of patients with HF in our geographical area, serum vitamin D levels correlated with the solar radiation received during the 2–3 months prior to the fracture, while the seasonal pattern of HF was inversely proportional to the seasonality of vitamin D levels [43]. Finally, a recent Japanese study exploring the external causes of death from 1979 to 2015 in relation to ambient temperature found that both cold and heat exposures were associated with increased falls-related deaths [44]. In view of all the above, we hypothesize that gradual increase in solar radiation with a cumulative effect on bone mineralization, along with good visibility and pleasant temperatures without extreme heat, would have led to a minimum HFi in June and the opposite trend in December.

In addition to sunshine, only relative humidity showed a protective effect in general, and in all subgroups (except in people ≤ 75 years of age), in the regression model, which was more pronounced in people over 85 years of age. There are few studies that have analyzed the relationship between relative humidity and the risk of HF. In one aforementioned study performed in Spain, the authors described a different pattern in people under 75 years of age, in which high relative humidity was associated with a higher HFi compared to older people, in whom the effect was almost null [13]. Another study from Israel involving subjects with a mean age of 78 years [45] did not find any associations. The rest of the parameters (atmospheric pressure and average wind speed) similarly failed to show any consistent association.

There are certain limitations in our study that deserve some mention. Firstly, the data provided comes from administrative sources that may be subject to notification and registration errors. Local privately owned hospitals do not have incentives to record discharge information in a systematic manner, which could lead to under-registration in some cases. Nevertheless, as Catalonia has universal and free access to public healthcare system, only a minimal percentage of HFs are admitted to private centers [46]. In addition, the relationship between climatic variables and fracture rates are temporal in nature and could be influenced by other uncontrolled factors, such as differences in mobility and clothing between seasons [39]. In fact, the ecological design of the study could lead to an ecological fallacy—the possibility of making incorrect conclusions about individual-level associations when only using aggregated data. Another limitation of our study is that we lack other important information related to the individual fracture risk: the mechanism of fracture or the place where the fracture occurred (indoors or outdoors). Finally, as we chose to analyze the time series using periods of one month, we believe that the effects of meteorological phenomena such as wind speed or icy days, which have immediate effects on fracture risk and are scarcely present in our environment, could be underestimated.

In summary, the data that we present from southern Europe show a decreasing trend in the rate of fractures in our population, especially in elderly women, until 2019. After the start of the Covid-19 pandemic, most studies from different geographical areas, including ours, reported a sharper decrease in the HFi and other FFs [47, 48]. We observed a marked seasonality in incidence rates, with a peak in autumn and a nadir in summer, especially in older people. Based on our data, insolation could be more responsible than temperature for the fluctuation of HFi in our country.

### Supplementary Information

Below is the link to the electronic supplementary material.

Supplementary file1 (DOCX 368 kb)

## Data Availability

The data that support the findings of this study are partly available in the supplementary material of this article. Other data will be available from the corresponding author upon reasonable request.
