# Supplementary material for: Trend and Seasonality of Hip Fractures in Catalonia, Spain: Exploring the Influence of Climate
Source: Calcif Tissue Int. 2024 Feb 10;114(4):326–39. doi: 10.1007/s00223-024-01182-8 (PMC10957628; doi:10.1007/s00223-024-01182-8)
Supplement: Supplementary file 1 — Supplementary file1 (DOCX 368 kb) [file 223_2024_1182_MOESM1_ESM.docx]

Supplementary Material

Table S1: Mean (SD) monthly average values of the meteorological parameters, by season, in Catalonia during the period 2010-2019 (meteorological stations level below 1,500 meters of altitude)

|  | Winter | Spring | Summer | Autumn | Overall |
| --- | --- | --- | --- | --- | --- |
| Daily insolation (MJ/m^2^) | 11.45(0.96) | 22.81(0.98) | 21.76(0.68) | 8.96(0.41) | 16.24(6.24) |
| Temperature (ºC) | 7.62(0.65) | 16.46(0.90) | 22.14(0.48) | 10.91(0.72) | 14.28(5.64) |
| Rainy days (number) | 9.15(2.76) | 9.15(1.61) | 6.73(1.62) | 10.12(2.33) | 8.79(2.41) |
| Icy days (number) | 8.73(2.06) | 0.34(0.18) | 0.01(0.01) | 4.40(2.01) | 3.37(3.85) |
| Atmospheric pressure (hPa) | 972.18(3.79) | 971.55(1.64) | 973.24(1.09) | 973.63(3.54) | 972.65(2.79) |
| Relative humidity (%) | 69.80(3.54) | 65.86(2.52) | 65.70(2.65) | 76.16(3.50) | 69.38(5.2) |
| Wind speed (m/s) | 2.64(0.18) | 2.47(0.13) | 2.28(0.09) | 2.22(0.21) | 2.4(0.23) |

MJ; Megajoules, hPa; Hectopascals

Table S2: Crude incidence rate of HF in Catalonia according to sex, age and type of fracture (x 100,000 inhabitants ≥ 65 years) and % change from 2010 to 2019.

|  | Crude incidence rate (x100,000) | | | | | | | | | | | | | | | | | | | | | | | | | | |
| --- | --- | --- | --- | --- | --- | --- | --- | --- | --- | --- | --- | --- | --- | --- | --- | --- | --- | --- | --- | --- | --- | --- | --- | --- | --- | --- | --- |
| Year | Overall | | | Women | | | Men | | | 65-74 years | | | 75-84 years | | | ≥ 85 years | | | | Extracapsular fractures | | | | Intracapsular fractures | | | |
|  | CR | UCI | LCI | CR | UCI | LCI | CR | UCI | LCI | CR | UCI | LCI | CR | UCI | LCI | CR | UCI | LCI | CR | | UCI | LCI | CR | | UCI | LCI |  |
| 2010 | **697.7** | 712.4 | 683.1 | **900.2** | 922.0 | 878.4 | **418.6** | 436.1 | 401.2 | **140.8** | 150.2 | 131.3 | **756.9** | 781.7 | 732.2 | **2455.3** | 2528.7 | 2382.0 | **382.4** | | 393.2 | 371.5 | **315.4** | | 325.2 | 305.5 |  |
| 2011 | **675.2** | 689.5 | 660.9 | **877.7** | 899.1 | 856.2 | **397.6** | 414.5 | 380.7 | **127.9** | 137.0 | 118.9 | **719.5** | 743.6 | 695.5 | **2367.8** | 2438.2 | 2297.3 | **368.0** | | 378.5 | 357.4 | **307.2** | | 316.9 | 297.6 |  |
| 2012 | **672.4** | 686.5 | 658.2 | **878.7** | 899.9 | 857.4 | **391.3** | 407.9 | 374.7 | **124.8** | 133.7 | 116.0 | **710.6** | 734.3 | 686.9 | **2309.9** | 2377.6 | 2242.2 | **373.0** | | 383.5 | 362.5 | **299.4** | | 308.8 | 290.0 |  |
| 2013 | **675.2** | 689.3 | 661.1 | **881.2** | 902.4 | 860.0 | **395.7** | 412.2 | 379.2 | **132.6** | 141.7 | 123.6 | **702.7** | 726.2 | 679.1 | **2316.3** | 2383.2 | 2249.3 | **369.7** | | 380.1 | 359.3 | **305.5** | | 314.9 | 296.0 |  |
| 2014 | **652.9** | 666.5 | 639.2 | **846.4** | 867.0 | 825.9 | **390.9** | 407.2 | 374.7 | **129.1** | 137.8 | 120.4 | **661.6** | 684.7 | 638.6 | **2298.8** | 2364.4 | 2233.3 | **358.1** | | 368.2 | 347.9 | **294.8** | | 304.0 | 285.6 |  |
| 2015 | **688.3** | 702.2 | 674.3 | **890.9** | 911.8 | 869.9 | **415.4** | 432.0 | 398.8 | **138.8** | 147.6 | 130.0 | **722.5** | 747.1 | 698.0 | **2383.5** | 2449.1 | 2317.9 | **377.5** | | 387.9 | 367.2 | **310.8** | | 320.1 | 301.4 |  |
| 2016 | **694.1** | 708.1 | 680.2 | **894.5** | 915.4 | 873.6 | **425.1** | 441.8 | 408.4 | **141.6** | 150.5 | 132.7 | **703.3** | 727.3 | 679.2 | **2394.5** | 2459.1 | 2329.9 | **387.6** | | 398.0 | 377.2 | **306.5** | | 315.8 | 297.3 |  |
| 2017 | **672.1** | 685.7 | 658.5 | **856.8** | 877.1 | 836.5 | **424.7** | 441.2 | 408.1 | **139.4** | 148.1 | 130.7 | **648.0** | 671.2 | 624.8 | **2356.9** | 2419.9 | 2294.0 | **364.9** | | 374.9 | 354.8 | **307.2** | | 316.4 | 298.0 |  |
| 2018 | **637.1** | 650.2 | 624.0 | **808.4** | 828.0 | 788.8 | **408.3** | 424.4 | 392.2 | **144.2** | 152.9 | 135.4 | **631.2** | 654.1 | 608.2 | **2150.6** | 2209.7 | 2091.5 | **352.7** | | 362.5 | 343.0 | **284.4** | | 293.1 | 275.6 |  |
| 2019 | **646.5** | 659.6 | 633.3 | **825.6** | 845.2 | 805.9 | **407.9** | 423.8 | 391.9 | **138.9** | 147.5 | 130.4 | **615.1** | 637.7 | 592.6 | **2229.7** | 2289.0 | 2170.3 | **356.1** | | 365.8 | 346.3 | **290.4** | | 299.2 | 281.6 |  |
| Change (%) between 2010 and 2019 | -7.3 |  |  | -8.3 |  |  | -2.6 |  |  | -1.3 |  |  | -18.7 |  |  | -9.2 |  |  | -6.9 | |  |  | -8.0 | |  |  |  |

LCI: lower confidence interval, UCI: upper confidence interval

Table S3: Age-standardised incidence rate of HF in Catalonia according to sex, age and type of fracture (x 100,000 inhabitants ≥ 65 years) and % change from 2010 to 2019

|  | Standardized incidence rate (x100,000) | | | | | | | | |
| --- | --- | --- | --- | --- | --- | --- | --- | --- | --- |
| Year | Overall | | | Women | | | Men | | |
|  | SR | UCI | LCI | SR | UCI | LCI | SR | UCI | LCI |
| 2010 | **728.1** | 742.5 | 713.6 | **935.0** | 956.6 | 913.4 | **440.3** | 457.6 | 423.1 |
| 2011 | **691.9** | 706.0 | 677.8 | **895.3** | 916.5 | 874.2 | **411.6** | 428.3 | 394.9 |
| 2012 | **677.3** | 691.3 | 663.4 | **883.1** | 904.1 | 862.1 | **396.0** | 412.4 | 379.7 |
| 2013 | **677.1** | 691.0 | 663.1 | **882.7** | 903.7 | 861.7 | **397.5** | 413.9 | 381.1 |
| 2014 | **652.9** | 666.5 | 639.2 | **846.4** | 867.0 | 825.9 | **390.9** | 407.2 | 374.7 |
| 2015 | **685.1** | 699.1 | 671.0 | **888.0** | 909.0 | 866.9 | **412.3** | 428.9 | 395.6 |
| 2016 | **682.5** | 696.5 | 668.5 | **882.8** | 903.8 | 861.8 | **415.3** | 432.1 | 398.6 |
| 2017 | **652.2** | 665.9 | 638.5 | **836.6** | 857.1 | 816.2 | **407.0** | 423.6 | 390.5 |
| 2018 | **616.4** | 629.7 | 603.1 | **788.2** | 808.1 | 768.4 | **389.8** | 406.0 | 373.5 |
| 2019 | **624.5** | 637.8 | 611.1 | **804.1** | 824.1 | 784.0 | **388.4** | 404.6 | 372.2 |
| Change (%) between 2010 and 2019 | -14.2 |  |  | -14.0 |  |  | -11.8 |  |  |

LCI: lower confidence interval, UCI: upper confidence interval

Fig S1: Monthly HFi by sex, age groups and anatomical site


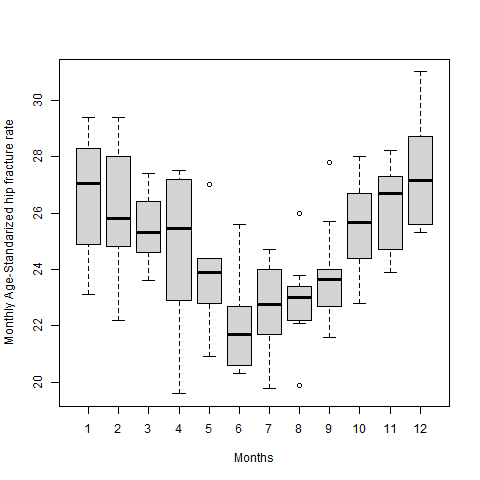

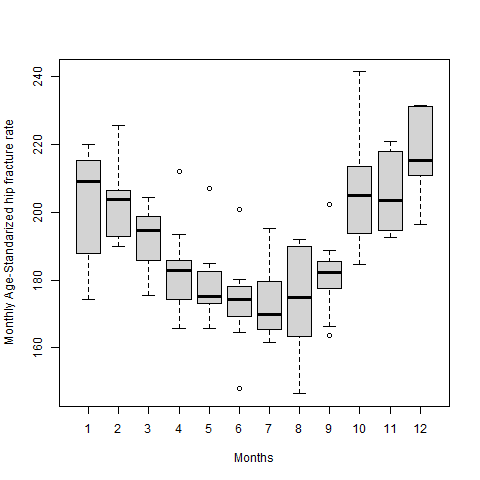


Intracapsular fractures

Extracapsular fractures


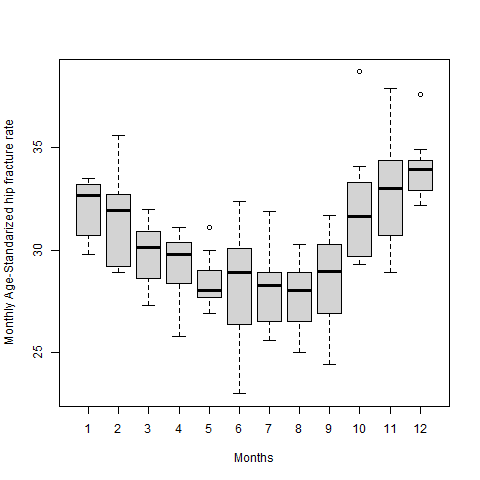

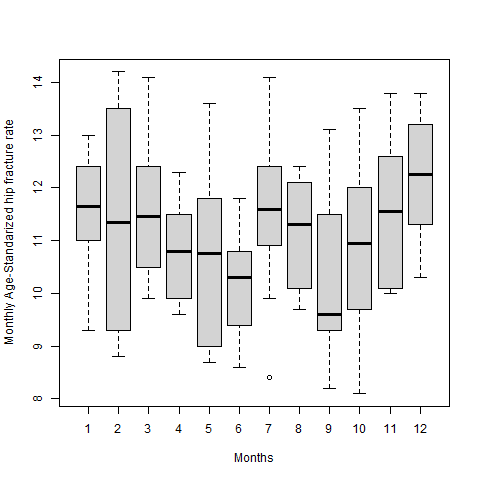

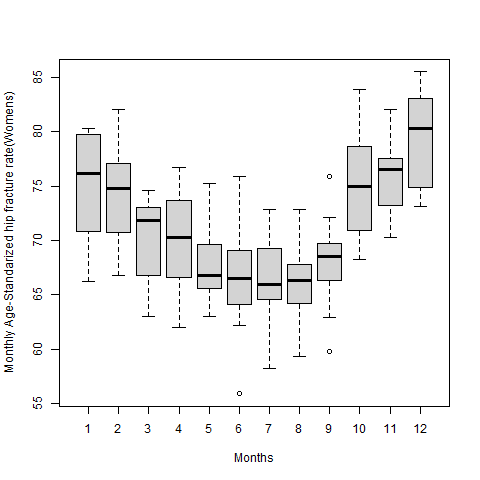

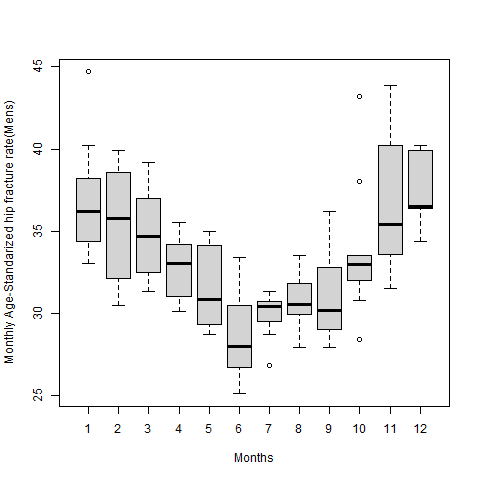


Women

Men

75-84 years

65-74 years


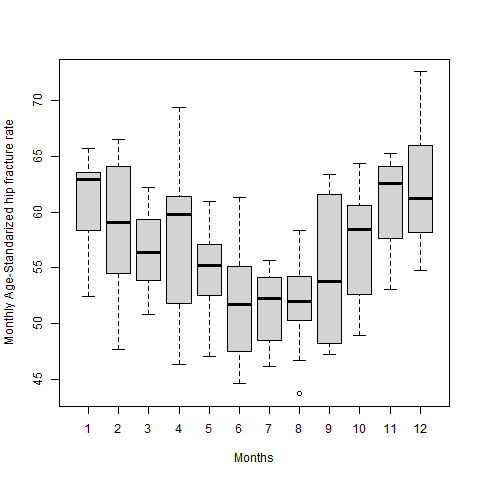


≥ 85 years

Table S3: Comparison of monthly average (SD) insolation, average (SD) temperature and hip fracture incidence rate in the overall population aged ≥ 65 years in Catalonia during the period 2010-2019 (months adjusted to 30 days)

| Calendar Month | Solar radiation (MJ/m^2^) | Temperature (ºC) | Overall standardized HFi x 100.000 people aged ≥ 65 years |
| --- | --- | --- | --- |
| January | 7.5 (0.6) | 6.2 (1.2) | 58.7 (3.4) |
| February | 11.1 (1.3) | 6.8 (1.5) | 57.9 (4.0) |
| March | 15.8 (1.4) | 9.9 (0.9) | 55.3 (2.3) |
| April | 19.5 (1.4) | 12.8 (1.0) | 54.0 (3.3) |
| May | 23.4 (1.4) | 15.9 (1.4) | 52.1 (2.5) |
| June | 25.8 (0.9) | 20.6 (1.2) | 50.3 (3.8) |
| July | 25.7 (0.8) | 23.5 (1.2) | 50.7 (2.8) |
| August | 22.4 (0.9) | 23.2 (0.8) | 50.8 (2.8) |
| September | 17.3 (1.0) | 19.7 (0.1) | 52.3 (3.4) |
| October | 12.2 (0.9) | 15.7 (1.0) | 57.7 (4.3) |
| November | 8.0 (0.1) | 10.1 (1.0) | 59.1 (3.1) |
| December | 6.6 (0.4) | 6.9 (1.1) | 61.6 (3.2) |

Figure S2: Decomposition of the additive time series in the trend, the seasonal component and the residuals in the Seasonal-ARIMA analysis


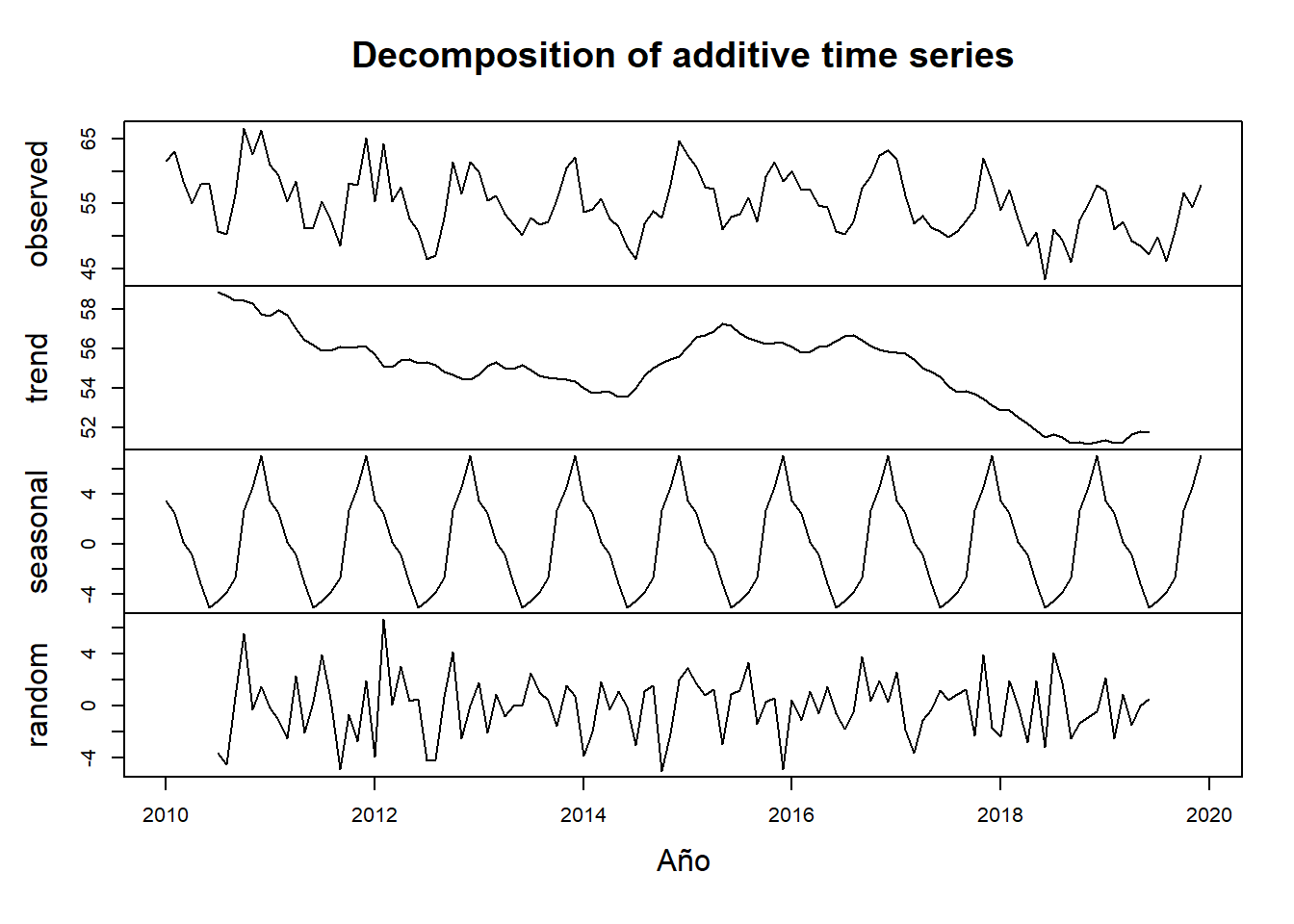


year

Fig S3: Representation of the prediction model based on GAM considering insolation, temperature, relative humidity,

Age-standardised hip fracture incidence rate

icy days, and rainy days as explicative variables.


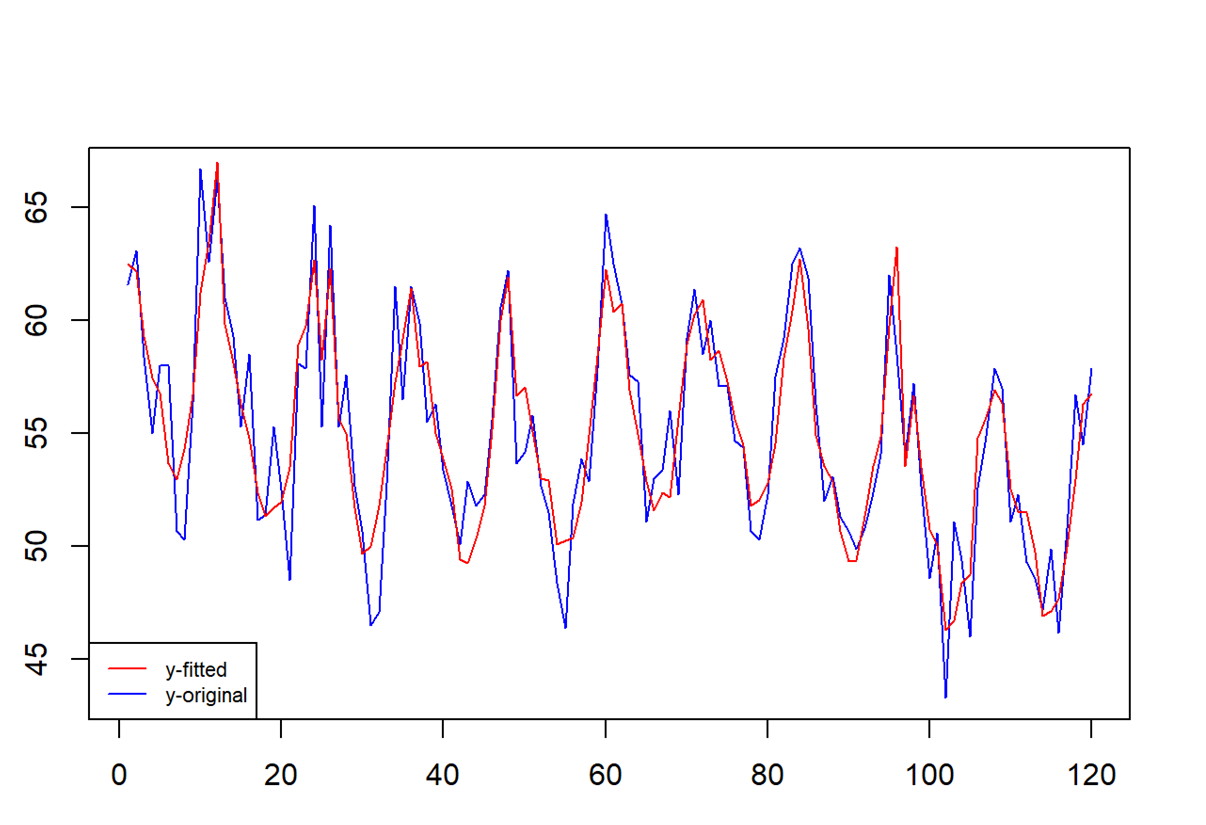


Time series (months)
